# When to Start Antiretroviral Therapy in Children Aged 2–5 Years: A Collaborative Causal Modelling Analysis of Cohort Studies from Southern Africa

**DOI:** 10.1371/journal.pmed.1001555

**Published:** 2013-11-19

**Authors:** Michael Schomaker, Matthias Egger, James Ndirangu, Sam Phiri, Harry Moultrie, Karl Technau, Vivian Cox, Janet Giddy, Cleophas Chimbetete, Robin Wood, Thomas Gsponer, Carolyn Bolton Moore, Helena Rabie, Brian Eley, Lulu Muhe, Martina Penazzato, Shaffiq Essajee, Olivia Keiser, Mary-Ann Davies, for the International Epidemiologic Databases to Evaluate AIDS–Southern Africa (IeDEA-SA) Collaboration

**Affiliations:** 1 Centre for Infectious Disease Epidemiology and Research, University of Cape Town, Cape Town, South Africa; 2 Institute of Social and Preventive Medicine, University of Bern, Bern, Switzerland; 3 Africa Centre for Health and Population Studies, University of KwaZulu-Natal, Somkhele, South Africa; 4 Lighthouse Trust Clinic, Kamuzu Central Hospital, Lilongwe, Malawi; 5 Liverpool School of Tropical Medicine, Liverpool, United Kingdom; 6 Wits Reproductive Health and HIV Institute, Harriet Shezi Children's Clinic, Chris Hani Baragwanath Hospital, Soweto, South Africa; 7 Faculty of Health Sciences, University of Witwatersrand, Johannesburg, South Africa; 8 Empilweni Service and Research Unit, Rahima Moosa Mother and Child Hospital and University of Witwatersrand, Johannesburg, South Africa; 9 Médecins Sans Frontières South Africa, Cape Town, South Africa; 10 Khayelitsha ART Programme, Khayelitsha, Cape Town, South Africa; 11 Sinikithemba Clinic, McCord Hospital, Durban, South Africa; 12 Newlands Clinic, Harare, Zimbabwe; 13 Desmond Tutu HIV Centre, Cape Town, South Africa; 14 Institute of Infectious Diseases and Molecular Medicine, University of Cape Town, Cape Town, South Africa; 15 University of North Carolina at Chapel Hill, Chapel Hill, North Carolina, United States of America; 16 Centre for Infectious Disease Research in Zambia, Lusaka, Zambia; 17 Tygerberg Academic Hospital, University of Stellenbosch, Stellenbosch, South Africa; 18 School of Child and Adolescent Health, Red Cross War Memorial Children's Hospital, University of Cape Town, Cape Town, South Africa; 19 Department of Maternal, Child and Adolescent Health, World Health Organization, Geneva, Switzerland; 20 Clinical Trial Unit, Medical Research Council, London, United Kingdom; 21 Clinton Health Access Initiative, Boston, Massachusetts, United States of America; San Francisco General Hospital, United States of America

## Abstract

**Background:**

There is limited evidence on the optimal timing of antiretroviral therapy (ART) initiation in children 2–5 y of age. We conducted a causal modelling analysis using the International Epidemiologic Databases to Evaluate AIDS–Southern Africa (IeDEA-SA) collaborative dataset to determine the difference in mortality when starting ART in children aged 2–5 y immediately (irrespective of CD4 criteria), as recommended in the World Health Organization (WHO) 2013 guidelines, compared to deferring to lower CD4 thresholds, for example, the WHO 2010 recommended threshold of CD4 count <750 cells/mm^3^ or CD4 percentage (CD4%) <25%.

**Methods and Findings:**

ART-naïve children enrolling in HIV care at IeDEA-SA sites who were between 24 and 59 mo of age at first visit and with ≥1 visit prior to ART initiation and ≥1 follow-up visit were included. We estimated mortality for ART initiation at different CD4 thresholds for up to 3 y using g-computation, adjusting for measured time-dependent confounding of CD4 percent, CD4 count, and weight-for-age *z*-score. Confidence intervals were constructed using bootstrapping.

The median (first; third quartile) age at first visit of 2,934 children (51% male) included in the analysis was 3.3 y (2.6; 4.1), with a median (first; third quartile) CD4 count of 592 cells/mm^3^ (356; 895) and median (first; third quartile) CD4% of 16% (10%; 23%). The estimated cumulative mortality after 3 y for ART initiation at different CD4 thresholds ranged from 3.4% (95% CI: 2.1–6.5) (no ART) to 2.1% (95% CI: 1.3%–3.5%) (ART irrespective of CD4 value). Estimated mortality was overall higher when initiating ART at lower CD4 values or not at all. There was no mortality difference between starting ART immediately, irrespective of CD4 value, and ART initiation at the WHO 2010 recommended threshold of CD4 count <750 cells/mm^3^ or CD4% <25%, with mortality estimates of 2.1% (95% CI: 1.3%–3.5%) and 2.2% (95% CI: 1.4%–3.5%) after 3 y, respectively. The analysis was limited by loss to follow-up and the unavailability of WHO staging data.

**Conclusions:**

The results indicate no mortality difference for up to 3 y between ART initiation irrespective of CD4 value and ART initiation at a threshold of CD4 count <750 cells/mm^3^ or CD4% <25%, but there are overall higher point estimates for mortality when ART is initiated at lower CD4 values.

*Please see later in the article for the Editors' Summary*

## Abstract 2

Michael Schomaker and colleagues estimate the mortality associated with starting ART at different CD4 thresholds among children aged 2–5 years using observational data collected in cohort studies in Southern Africa.

*Please see later in the article for the Editors' Summary*

## Editors' Summary

**Background:**

Infection with HIV, the virus that causes AIDS, contributes substantially to the burden of disease in children. Worldwide, more than 3 million children younger than 15 years old (90% of whom live in sub-Saharan Africa) are HIV-positive, and every year around 330,000 more children are infected with HIV. Children usually acquire HIV from their mother during pregnancy, birth, or breastfeeding. The virus gradually destroys CD4 lymphocytes and other immune system cells, leaving infected children susceptible to other potentially life-threatening infections. HIV infection can be kept in check, with antiretroviral therapy (ART)—cocktails of drugs that have to be taken daily throughout life. ART is very effective in children but is expensive, and despite concerted international efforts over the past decade to provide universal access to ART, in 2011, less than a third of children who needed ART were receiving it.

**Why Was This Study Done?:**

For children diagnosed as HIV-positive between the ages of two and five years, the 2010 World Health Organization (WHO) guidelines for the treatment of HIV infection recommended that ART be initiated when the CD4 count dropped below 750 cells/mm^3^ blood or when CD4 cells represented less than 25% of the total lymphocyte population (CD4 percent). Since June 2013, however, WHO has recommended that all HIV-positive children in this age group begin ART immediately, irrespective of their CD4 values. Earlier ART initiation might reduce mortality (death) and morbidity (illness), but it could also increase the risk of toxicity and of earlier development of drug resistance. In this causal modeling analysis, the researchers estimate the mortality associated with starting ART at different CD4 thresholds among children aged 2–5 years using observational data collected in cohort studies of ART undertaken in southern Africa. Specifically, they compared the estimated mortality associated with the WHO 2010 and WHO 2013 guidelines. Observational studies compare the outcomes of groups (cohorts) with different interventions (here, the timing of ART initiation). Data from such studies are affected by time-dependent confounding: CD4 count, for example, varies with time and is a predictor of both ART initiation and the probability of death. Causal modeling techniques take time-dependent confounding into account and enable the estimation of the causal effect of an intervention on an outcome from observational data.

**What Did the Researchers Do and Find?:**

The researchers used g-computation (a type of causal modeling) adjusting for time-dependent confounding of CD4 percent, CD4 count, and weight-for-age *z*-score (a measure of whether a child is underweight for their age that provides a proxy indicator of the clinical stage of HIV infection) to estimate mortality for ART initiation at different CD4 thresholds in 2,934 ART-naïve, HIV-positive children aged 2–5 years old at their first visit to one of eight study sites in southern Africa. The average initial CD4 values of these children were a CD4 count of 592 cells/mm^3^ and a CD4 percent of 16%. The estimated cumulative mortality after three years was 3.4% in all children if ART was never started. If all children had started ART immediately after diagnosis irrespective of CD4 value or if the 2010 WHO-recommended threshold of a CD4 count below 750 cells/mm^3^ or a CD4 percent below 25% was followed, the estimated cumulative mortalities after three years were 2.1% and 2.2%, respectively (a statistically non-significant difference).

**What Do These Findings Mean?:**

These findings suggest that, among southern African children aged 2–5 years at HIV diagnosis, there is no difference in mortality for up to three years between children in whom ART is initiated immediately and those in whom ART initiation is deferred until their CD4 value falls below a CD4 count of 750 cells/mm^3^ or a CD4 percent of 25%. Although causal modeling was used in this analysis, the accuracy of these results may be affected by residual confounding. For example, the researchers were unable to adjust for the clinical stage of HIV disease at HIV diagnosis and instead had to use weight-for-age *z*-scores as a proxy indicator of disease severity. Other limitations of the study include the large number of children lost to follow-up and a possible lack of generalizability—most of the study participants were from urban settings in South Africa. Importantly, however, these findings suggest that the recent change in the WHO guidelines for ART initiation in young children is unlikely to increase or reduce mortality, with the proviso that the long-term effects of earlier ART initiation such as toxicity and the development of resistance to ART need to be explored further.

**Additional Information:**

Please access these websites via the online version of this summary at http://dx.doi.org/10.1371/journal.pmed.1001555

- Information is available from the US National Institute of Allergy and Infectious Diseases on HIV infection and AIDS
- NAM/aidsmap provides basic information about HIV/AIDS and summaries of recent research findings on HIV care and treatment
- Information is available from Avert, an international AIDS charity, on many aspects of HIV/AIDS, including information on HIV and AIDS in Africa and on children and HIV/AIDS (in English and Spanish)
- The UNAIDS World AIDS Day Report 2012 provides up-to-date information about the AIDS epidemic and efforts to halt it; the 2013 Progress Report on the Global Plan provides information on progress towards eliminating new HIV infections among children by 2015
- The World Health Organization provides information about universal access to AIDS treatment (in several languages); its 2010 guidelines for ART in infants and children and its 2013 consolidated guidelines on the use of ART can be downloaded
- The researchers involved in this study are part of the International Epidemiologic Databases to Evaluate AIDSSouthern Africa collaboration, which develops and implements methodology to generate the large datasets needed to address high-priority research questions related to HIV/AIDS
- Personal stories about living with HIV/AIDS, including stories from young people infected with HIV, are available through Avert, through NAM/aidsmap, and through the charity website Healthtalkonline

## Introduction

HIV infection continues to contribute substantially to the burden of disease in children, with an estimated 330,000 new paediatric infections worldwide in 2011. Paediatric antiretroviral combination therapy (ART) is highly effective, but ART coverage for children who need it was below 30% in 2011 [1]. Furthermore, there is still limited evidence from randomized controlled trials (RCTs) regarding the optimal timing of ART initiation in children aged 2–5 y [2],[3]. While earlier ART initiation may reduce mortality and morbidity, it could also increase the risk of toxicity and earlier development of drug resistance.

The Children with HIV Early Antiretroviral Therapy trial demonstrated a 76% (95% CI: 49%–89%) reduction in mortality associated with immediate compared to deferred ART in infants less than 3 mo of age; however, results of studies in older children are less clear [4]–[6]. The Pediatric Randomized to Early versus Deferred Initiation in Cambodia and Thailand (PREDICT) trial [7]–[9] enrolled children aged 1–12 y with initial CD4 percentage (CD4%) values of 15%–24% without US Centers for Disease Control and Prevention (CDC) category C disease. These children were randomly assigned to start ART immediately or defer therapy until their CD4% dropped below 15% or a CDC category C event occurred. There was no difference between the two arms in mortality, AIDS-free survival, number of new category B and C events, neurodevelopmental outcomes, rate of hospital admission, or number of drug-related adverse events [7],[9]. However, less than a third of the children included in the trial (96 in total) were in the 2–5-y age group. This group may experience more rapid disease progression than older children in the absence of ART, and so the benefits of immediate ART might be greater [10]–[12]. In addition, because of the low event rate for the primary outcomes (five CDC category C events, one death), the PREDICT study authors concluded that the study was underpowered to detect a difference between the two randomization groups [9].

In the absence of evidence from RCTs, observational data from cohort studies may be used to address this question. Such analyses are not straightforward, however, because of time-dependent confounding. The CD4 count, for example, is a time-dependent confounder that varies with time, predicts the initiation of ART, but also predicts the probability of dying. Causal modelling techniques such as g-computation enable estimation of the causal effect of an intervention on an outcome, taking into account such time-dependent confounding [13]–[15]. We used data from the International Epidemiologic Databases to Evaluate AIDS–Southern Africa (IeDEA-SA) collaboration to estimate mortality for up to 3 y of follow-up associated with starting ART at a range of different CD4 values in children aged 2–5 y. Our primary study aim was to determine whether there is any difference in estimated mortality associated with starting ART at the World Health Organization (WHO) 2010 recommended threshold of CD4 count <750 cells/mm^3^ or CD4% <25% [16] in comparison to starting ART immediately as recommended in the WHO 2013 guidelines.

## Methods

### Ethics Statement

The IeDEA-SA collaboration study was approved by the University of Cape Town and University of Bern human research ethics committees. The requirement for informed consent was waived, as only anonymized data that were already collected as part of routine monitoring contributed to the collaborative dataset.

### Cohort Description and Selection of Children

This study is based on HIV treatment cohorts in southern Africa that took part in the IeDEA-SA collaboration (http://www.iedea-sa.org/). The collaboration has been described in detail elsewhere [17],[18]. In brief, data were collected at each site as part of routine monitoring and were transferred to data centres at the University of Cape Town, South Africa, or the University of Bern, Switzerland, at regular intervals. All programmes obtained ethical approval from the relevant local institutions before contributing anonymized patient data to IeDEA-SA. The present analysis is restricted to eight cohorts that routinely captured both pre-ART data and post-ART data of HIV-infected children: Lighthouse Trust Clinic in Malawi; Newlands Clinic in Zimbabwe; and McCord Hospital, Harriet Shezi Children's Clinic, Gugulethu Community Health Centre, Khayelitsha ART Programme, Hlabisa HIV Treatment and Care Programme, and Rahima Moosa Mother and Child Hospital in South Africa. All clinics except McCord Hospital were public health sector programmes with research support providing primary care to urban populations, but rural populations were included in the Hlabisa HIV Treatment and Care Programme [18]. At Harriet Shezi Children's Clinic and Rahima Moosa Mother and Child Hospital, secondary and tertiary care are available in addition to primary care.

In South Africa, the recommended first-line regimen was two nucleoside reverse transcriptase inhibitors plus either lopinavir/ritonavir (children <3 y of age or <10 kg) or efavirenz (children >3 y of age and >10 kg). In Malawi and Zimbabwe the recommended first-line regimen for all children was two nucleoside reverse transcriptase inhibitors plus nevirapine. The nucleoside reverse transcriptase inhibitors used were mainly stavudine or zidovudine plus lamivudine.

**Figure 1:**
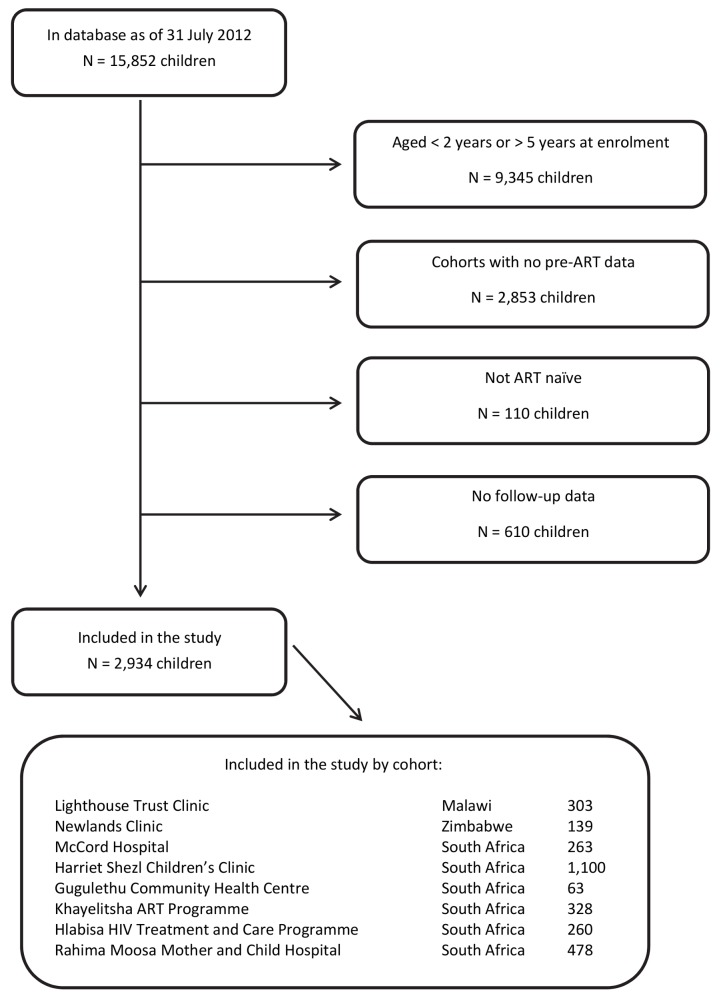
Flowchart: selection of patients.

All children who were at least 24 mo of age at enrolment into HIV care, enrolled between 1 January 2000 and 30 June 2012, and were not older than 60 mo were included. Children who were not ART-naïve at their time of entry into the HIV care programmes or who had no follow-up data were excluded from the analysis. The detailed process of selection of children eligible for this analysis is provided in Figure 1.

### Variable Definitions and Loss to Follow-Up

Baseline characteristics included CD4 count, CD4%, weight-for-age *z*-score (WAZ), and height-for-age *z*-score (HAZ), as well as the child's sex and age at first visit. All *z*-scores were calculated using WHO standards [19]. Follow-up data consisted of CD4 count, CD4%, and WAZ, evaluated 1 mo after the first clinic visit and in 3-mo intervals starting 3 mo after the first visit, for up to 36 mo in care. If two or more observations per interval were available, the one closest to the middle of the interval was used. The date of ART initiation (if applicable) was captured as well. If there were no data for a child in a particular time interval, data were defined to be missing. The outcome was mortality and was ascertained by the individual cohorts. Children were defined as being lost to follow-up (LTFU) and were censored if they had no contact with their health care facility for 9 mo before database closure (visits are scheduled up to 6 mo apart and clinic visits being delayed for up to 3 mo are possible).

### Multiple Imputation of Missing Baseline and Follow-Up Data

To account for missing follow-up data for CD4 count, CD4%, and WAZ, a child's last observation was carried forward for a maximum of 9 mo. For the remaining missing follow-up data, as well as missing baseline data for CD4 count, CD4%, WAZ, and HAZ, longitudinal multiple imputation by means of the expectation maximisation bootstrap algorithm [20] was used to create ten imputed datasets. Imputations were utilised only after 9 mo without any visit data, as from there on it is plausible that CD4/WAZ measurements that determine treatment assignment were taken (and are thus needed to adjust for time-dependent confounding) but not electronically recorded, probably because of administrative or clerical errors. The missing at random assumption is therefore likely to hold. The imputation model included all measured baseline and follow-up variables, mortality, follow-up time, a variable indicating which observations were carried forward, and the region (Zimbabwe, Malawi, South Africa urban, South Africa rural). The longitudinal structure of the data was explicitly considered in the expectation maximisation bootstrap algorithm, nonlinear time trends were allowed, and lag and lead variables of CD4 count, CD4%, and HAZ were added to the imputation model. All results of the multivariate analysis are based on the imputed datasets and were combined using Rubin's rules [21].

### Statistical Analyses

Baseline characteristics were described using proportions or medians (reported with first; third quartile) overall and for different ART initiation thresholds. Follow-up data were described at 12 mo and 24 mo after the first clinic visit.

We then estimated mortality over 3 y of follow-up for ART initiation at different CD4 thresholds using causal modelling (g-computation) [13], adjusting for time-dependent confounding of CD4%, CD4 count, and WAZ, with 95% bootstrap confidence intervals based on 200 bootstrap samples. WAZ served as a proxy variable for WHO stage, which was not routinely available in our data: many clinical events that define advanced clinical WHO stage, and thus disease progression and severity, relate to a child's WAZ—for example, malnutrition, tuberculosis, persistent diarrhoea, severe wasting, recurrent bacterial pneumonia, recurrent bacterial infection, candidiasis, and chronic lung disease [22]–[24]. WAZ was therefore expected to approximately adjust for the time-dependent confounding induced by stage-defining severe clinical events. The analysis was also adjusted with respect to baseline CD4 count, CD4%, WAZ, HAZ, sex, age, and region.

The primary comparison we considered was (i) giving ART immediately irrespective of CD4 criteria or (ii) deferring ART until a child's CD4 cell count falls below 750 cells/mm^3^ or CD4% falls below 25%; further strategies we considered were (iii) deferring ART until the threshold of CD4 count <500 cells/mm^3^ or CD4% <20% is reached, (iv) deferring ART until the threshold of CD4 count <250 cells/mm^3^ or CD4% <15% is reached, and (v) no ART is given.

The details of our g-computation algorithm are contained in Box 1. Briefly, the observed associations in the data were used to simulate and evaluate counterfactual datasets that would have been observed under each of the above initiation rules. This mimics an RCT that compares the effect of the different initiation rules. These datasets were standardised with respect to the time-dependent confounders (CD4 count, CD4%, WAZ) that influence both mortality and ART initiation in children [25]. To implement this, we first modelled the association of these time-dependent confounders at all follow-up visits (at time *t* = 1, 3, 6,… mo) with disease progression history (follow-up data at time *t*−1), baseline characteristics (CD4 count, CD4%, WAZ), demographics (age, sex, region), and ART (at time *t*−1, included under an intention to treat assumption) using additive linear models. We used additive logistic regression to model the association of death with the same variables at each follow-up interval. For all these models nonlinear relationships and interactions with baseline characteristics were explored and included if they improved the generalised cross-validation score. In the second step we used the observed baseline data and predictions of the fitted models under a specific treatment initiation rule to simulate the time-dependent confounders and the outcome “forward in time”. These two steps were repeated for all the above specified ART initiation rules, and mortality in the simulated datasets was evaluated thereafter. All these steps were repeated for all bootstrap samples in each imputed dataset and combined appropriately.

**Box 1. Details of the g-Computation Algorithm**

**Step 1: Model fitting.** For the 3-y period of follow-up we considered the follow-up times *t* = 0, 1, 3, 6, 9,…, 36 mo after the first clinic visit.

- Time-dependent confounders. We used additive linear models to estimate the association of the time-dependent confounders (CD4 count, CD4%, WAZ) with
  - disease progression history (CD4 count, CD4%, and WAZ at time *t*−1)
  - baseline characteristics (CD4 count, CD4%, WAZ, HAZ)
  - demographics (age, sex, region)
  - the intervention (ART at time *t*−1)
  for *t* = 1, 3, 6,…, 36 mo. This corresponds to fitting three models (corresponding to the three time-dependent confounders) for 12 points in time.
- Outcome. We used a logistic additive model to estimate the association of the outcome (death) with the time-dependent confounders at time *t*, disease progression history, baseline characteristics, demographics, and intervention for *t* = 1, 3, 6,…, 36 mo. This corresponds to fitting one model for 12 points in time.
- Model building. The models were built as follows:
  - CD4 count was square root transformed in the models where CD4 count was the outcome variable.
  - Generalized-cross-validation-based model selection ensured a good bias–variance tradeoff of the models.
  - Nonlinear interactions of continuous variables (both time-dependent and baseline) with baseline characteristics (represented in categories) were explored to allow for flexible disease progression depending on how sick children present at their first visit. They were included if it improved the generalized cross-validation score of the model.
  - The effect of continuous variables on the outcomes was modelled in a nonlinear manner via *p*-splines. The complexity of the splines was determined by means of generalized cross-validation.

**Step 2: Simulation.** We simulated data for the children for one specific intervention *forward in time*.

At the first visit, *t* = 0, the data corresponded to the observed data of *all* children.

- Simulation of the covariates. For *t* = 1, 3, 6,…, 36 mo, CD4 count at time *t* was predicted by a random draw from a normal distribution where mean and standard error were obtained from the prediction of the additive linear model fitted in Step 1(a). The additive linear model corresponds to the model fitted at time *t*, and the intervention was set as specified by the treatment rule (e.g., ART = 0 if the intervention of interest was “never give ART”). Prediction for CD4% and WAZ were obtained in the same manner.
   The *simulated* values of CD4 count, CD4%, and WAZ at time *t*−1 were used when predicting the CD4 count, CD4%, and WAZ at time *t*.
- Simulation of the outcome. The hypothetical outcome was simulated based on a draw from a Bernoulli distribution with the probability obtained from the logistic additive model fitted in Step 1(b). Again, as in (a), we artificially set our dynamic intervention to the value corresponding to the strategy of interest. If the simulated outcome at time *t* was equal to 1 (death), then there was no more follow-up at time *t*+1.

**Step 3: Estimation of mortality.** We estimated the relative mortality for *t* = 1, 3, 6,…, 36 mo in evaluating our simulated dataset created in Step 2.

**Step 4: Intervention repetitions.** We repeated Steps 2 and 3 for all interventions of interest. These interventions are the following:

- Give a child ART immediately, irrespective of his/her CD4 count and CD4%
- Give a child ART when his/her absolute CD4 count falls below 750 cells/mm^3^ or his/her CD4% falls below 25%
- Give a child ART when his/her absolute CD4 count falls below 500 cells/mm^3^ or his/her CD4% falls below 20%
- Give a child ART when his/her absolute CD4 count falls below 250 cells/mm^3^ or his/her CD4% falls below 15%
- Never give a child ART

**Step 5: Bootstrap repetitions.** We repeated steps 1 to 4 for 200 bootstrap samples. The bootstrapping was utilized to obtain confidence intervals. The bounds of the 95% confidence intervals were set at the 2.5th and 97.5th percentiles of the distribution of the bootstrap estimates.

**Step 6: Multiple imputation.** Steps 1 to 5 were repeated for ten imputed sets of data. The multiple imputation was utilized with the Amelia II package in R. The imputation model included all measured variables and explicitly accounted for the longitudinal structure of the data.

**Diagnostics.** Imputation diagnostics (comparing imputed and observed densities, overimputation, convergence of expectation maximization chains, and time-series plots [41]) were evaluated to ensure the convergence of the algorithm and the appropriateness of the imputations.

We further conducted a sensitivity analysis, linking data from three of the South African cohorts (Hlabisa HIV Treatment and Care Programme, Khayelitsha ART Programme, McCord Hospital) with the national vital registry to obtain the vital status of children LTFU. We then imputed remaining events and survival times for children LTFU and unascertained [26] and estimated mortality using the g-computation algorithm described above.

We also calculated the percentage of children who presented with CD4 count above the threshold of 750 cells/mm^3^ or CD4% above 25% and estimated the probability of falling below one of these thresholds during the 3 y of follow-up using the Kaplan-Meier estimator. This analysis was restricted to pre-ART data, and we censored both children who died and children who were LTFU. The same probability was also estimated by means of cumulative incidence functions treating ART initiation, death, and LTFU as competing events. All analyses were conducted using R 2.15 [27].

## Results

From all 15,852 children in the database, there were 3,654 children in the eligible age range from cohorts with regular pre-ART data capturing. Of these, 110 children who were not ART-naïve and 610 ART-naïve children with no follow-up data were excluded. Most of the 2,934 children included in the study were from South Africa (85%; Figure 1). Median follow-up duration was 928 d (first; third quartile: 314; 1,082), during which 2,227 (75.9%) children started ART. In total there were 83 recorded deaths; 30 of these (36.1%) occurred in the first 3 mo, 40 (48.2%) in the first 6 mo. Overall, 662 (22.6%) children were defined to be LTFU.

The median (first; third quartile) age at enrolment into HIV care was 3.3 y (2.6; 4.1), and 1,501 (51.2%) were male (Table 1). The median CD4 count at enrolment was 592 cells/mm^3^ (356; 895), median CD4% was 16% (10; 23), and median WAZ and HAZ were −1.4 (−2.3; −0.5) and −2.6 (−3.5; −1.6), respectively. Among those who initiated ART, 1,716 (77%) started with a CD4 count below 750 cells/mm^3^ or CD4% below 25%, and 100 (4.5%) with a CD4 count above 750 cells/mm^3^ or CD4% above 25%. For 411 (18.5%) children, CD4 values at ART initiation were not available. The 707 (24.1%) children who never initiated ART had higher median CD4 count, CD4%, HAZ, and WAZ at their first clinic visit than children who initiated ART late (for example, at CD4 count below 750 cells/mm^3^ or CD4% below 25%) during follow-up. Among children who initiated ART, those who had lower CD4 counts or CD4% at ART initiation also had lower CD4 counts (and CD4%, WAZ, HAZ) at their first clinic visit (Table 1). Children who started ART had large CD4 and WAZ gains, up to >100%, compared to their first visit when presenting with a CD4 count below 250 cells/mm^3^ or CD4% below 15% at ART start (Table 2). The characteristics of children at enrolment were overall similar for the different cohorts, though there was some variation with respect to the median CD4 count (between 528 and 734 cells/mm^3^) and median WFA (between −0.9 and −1.8) (Table 3).

**Table 1:** Patient characteristics at first clinic visit overall (total) and stratified according to when and if ART was initiated.

Characteristic	CD4 Count/CD4% Threshold for ART				No ART	Unknown^a^	Total
	ART at <250 Cells/mm^3^ or <15%	ART at <500 Cells/mm^3^ or <20%	ART at <750 Cells/mm^3^ or <25%	ART at >750 Cells/mm^3^ and >25%			
**Sex**							
*n* (percent)	1,173 (100%)	352 (100%)	191 (100%)	100 (100%)	707 (100%)	411 (100%)	2,934 (100%)
Number male	624 (53.2%)	189 (53.7%)	90 (47.1%)	40 (40.0%)	347 (49.1%)	211 (51.3%)	1,501 (51.2%)
**Age (in years)**							
*n* (percent)	1,173 (100%)	352 (100%)	191 (100%)	100 (100%)	707 (100%)	411 (100%)	2,934 (100%)
Median (first; third quartile)	3.42 (2.65; 4.22)	3.21 (2.55; 4.05)	3.07 (2.45; 4.01)	3.05 (2.49; 3.85)	3.3 (2.57; 4.08)	3.20 (2.58; 4.11)	3.30 (2.59; 4.12)
**CD4 count**							
*n* (percent)	1,121 (95.57%)	333 (94.60%)	180 (94.24%)	94 (94.00%)	545 (77.09%)	214 (52.07%)	2,487 (84.76%)
Median (first; third quartile)	411 (228; 638)	665 (476; 910)	826.5 (661; 1,138)	1,107 (869; 1,426)	822 (547; 1,124)	549 (310; 819)	592 (356; 895)
**CD4%**							
*n* (percent)	1,114 (94.97%)	330 (93.75%)	176 (92.15%)	92 (92.00%)	483 (68.32%)	81 (19.71%)	2,276 (77.57%)
Median (first; third quartile)	11 (7.4; 14.3)	18 (16.6; 20.8)	22.55 (20.5; 25.0)	28.5 (25.5; 30.6)	23.9 (17.9; 29.2)	18 (10.4; 24.2)	16 (10.3; 23.0)
**WAZ**							
*n* (percent)	1,030 (87.81%)	301 (85.51%)	167 (87.43%)	85 (85.00%)	553 (78.22%)	382 (92.94%)	2,518 (85.82%)
Median (first; third quartile)	−1.46 (−2.45; −0.63)	−1.15 (−2.08; −0.39)	−1.27 (−2.06; −0.24)	−1.42 (−2.25; −0.70)	−1.12 (−2.07; −0.30)	−1.64 (−2.63; −0.86)	−1.37 (−2.32; −0.52)
**HAZ**							
*n* (percent)	856 (72.98%)	235 (66.76%)	128 (67.02%)	67 (67.00%)	458 (64.78%)	299 (72.75%)	2,043 (69.63%)
Median (first; third quartile)	−2.70 (−3.59; −1.83)	−2.35 (−3.29; −1.22)	−2.41 (−3.47; −1.44)	−2.61 (−3.22; −1.42)	−2.15 (−3.13; −1.23)	−2.76 (−3.67; −1.82)	−2.56 (−3.48; −1.59)

All initiation rules are mutually exclusive. For example, if a child started ART with a CD4 count of 650 cells/mm^3^ and a CD4% of 19%, then he/she would be included in the column “ART at <500 cells/mm^3^ or <20%” but no other column.

a Refers to children who initiated ART during their follow-up but did not have recorded data on both CD4% and CD4 count at the time of ART initiation.

**Table 2:** Evolution of CD4 count, CD4 percentage, and weight-for-age *z*-score over time from enrolment by ART initiation threshold, reported as median (first; third quartile).

Patient Characteristics (Stratified by CD4 Count/CD4% Threshold for ART)	Follow-Up Time		
	0 mo	12 mo	24 mo
**CD4 count**			
No ART	822 (547; 1,124)	923 (641; 1,192)	817 (634; 1,111)
ART at <250 cells/mm^3^ or <15%	411 (228; 638)	675 (422; 1,034)	869 (575; 1,209)
ART at <500 cells/mm^3^ or <20%	665 (476; 910)	869 (568; 1,288)	967 (663; 1,370)
ART at <750 cells/mm^3^ or <25%	827 (661; 1,138)	1,007 (733; 1,336)	1,029 (721; 1,457)
ART at >750 cells/mm^3^ and >25%	1,107 (869; 1,426)	1,284 (1,055; 1,643)	1,303 (1,081; 1,787)
Unknown	549 (310; 819)	846 (618; 1,191)	1,009 (720; 1,356)
Total	592 (356; 895)	814 (526; 1,170)	922 (639; 1,288)
**CD4%**			
No ART	23.9 (17.9; 29.2)	26.1 (21.0; 31.8)	26.7 (21.8; 33.6)
ART at <250 cells/mm^3^ or <15%	11.0 (7.4; 14.3)	18.0 (12.6; 24.3)	24.0 (18.2; 29.9)
ART at <500 cells/mm^3^ or <20%	18.0 (16.6; 20.8)	24.0 (19.5; 30.0)	27.0 (21.6; 34.0)
ART at <750 cells/mm^3^ or <25%	22.6 (20.5; 25.0)	26.0 (22.0; 31.9)	28.3 (23.0; 37.0)
ART at >750 cells/mm^3^ and >25%	28.5 (25.5; 30.6)	30.7 (27.6; 37.9)	34.8 (30.2; 41.9)
Unknown	18.0 (10.4; 24.2)	24.0 (18.5; 30.2)	28.8 (22.7; 35.0)
Total	16.0 (10.3; 23.0)	22.0 (15.8; 28.3)	26.0 (20.9; 32.5)
**WAZ**			
No ART	−1.12 (−2.07; −0.30)	−0.62 (−1.28; 0.08)	−0.47 (−1.08; 0.20)
ART at <250 cells/mm^3^ or <15%	−1.46 (−2.45; −0.63)	−1.00 (−1.72; −0.31)	−0.84 (−1.54; −0.21)
ART at <500 cells/mm^3^ or <20%	−1.15 (−2.08; −0.39)	−0.90 (−1.68; −0.19)	−0.89 (−1.46; −0.19)
ART at <750 cells/mm^3^ or <25%	−1.27 (−2.07; −0.24)	−0.86 (−1.60; −0.19)	−0.73 (−1.56; 0.05)
ART at >750 cells/mm^3^ and >25%	−1.42 (−2.25; −0.70)	−0.96 (−1.71; −0.37)	−0.99 (−1.50; −0.55)
Unknown	−1.65 (−2.63; −0.86)	−1.02 (−1.60; −0.27)	−0.81 (−1.41; −0.25)
Total	−1.37 (−2.32; −0.52)	−0.93 (−1.64; −0.25)	−0.80 (−1.48; −0.15)

**Table 3:** Patient characteristics at first clinic visit overall (total) and stratified according to cohort.

	Hlabisa HIV Treatment and Care Programme	Harriet Shezi Children's Clinic	McCord Hospital	Khayelitsha ART Programme	Rahima Moosa Mother and Child Hospital	Gugulethu Community Health Centre	Lighthouse Trust Clinic	Newlands Clinic	Total
**Sex**									
*n* (percent)	260 (100%)	1,100 (100%)	263 (100%)	328 (100%)	478 (100%)	63 (100%)	303 (100%)	139 (100%)	2,934 (100%)
Number male	130 (50.00%)	581 (52.82%)	137 (52.09%)	170 (51.83%)	241 (50.42%)	37 (58.73%)	142 (46.86%)	63 (45.32%)	1,501 (51.16%)
**Age (in years)**									
*n* (percent)	260 (100%)	1,100 (100%)	263 (100%)	328 (100%)	478 (100%)	63 (100%)	303 (100%)	139 (100%)	2,934 (100%)
Median(first; third quartile)	3.42 (2.69; 4.22)	3.31 (2.59; 4.17)	3.39 (2.63; 4.11)	3.11 (2.51; 3.96)	3.27 (2.58; 4.07)	3.42 (2.39; 4.47)	3.36 (2.63; 4.14)	3.21 (2.6; 4.14)	3.30 (2.59; 4.12)
**CD4 count**									
*n* (percent)	249 (95.77%)	947 (86.09%)	217 (82.51%)	221 (67.38%)	423 (88.49%)	62 (98.41%)	232 (76.57%)	136 (97.84%)	2,487 (84.76%)
Median (first; third quartile)	528 (294; 874)	574 (336; 871)	557 (347; 833)	734 (437; 1,024)	615 (350; 917)	587 (354; 796)	629 (403; 899)	538 (370; 813)	592 (355; 895)
**CD4%**									
*n* (percent)	239 (91.92%)	947 (86.09%)	216 (82.13%)	177 (53.96%)	422 (88.28%)	59 (93.65%)	204 (67.33%)	12 (8.63%)	2,276 (77.57%)
Median (first; third quartile)	16.0 (11.0; 23.0)	15.0 (9.0; 22.0)	17.0 (12.0; 24.0)	18.0 (13.0; 24.0)	15.8 (10.0; 22.7)	18.0 (11.0; 22.2)	18.5 (12.4; 24.4)	12.0 (9.3; 17.7)	16.0 (10.3; 23.0)
**WAZ**									
*n* (percent)	148 (56.92%)	1,075 (97.73%)	122 (46.39%)	314 (95.73%)	437 (91.42%)	0 (0%)	285 (94.06%)	137 (98.56%)	2,518 (85.82%)
Median (first; third quartile)	−1.20 (−2.04; −0.28)	−1.49 (−2.40; −0.70)	−0.90 (−1.75; −0.14)	−0.99 (−1.75; −0.04)	−1.29 (−2.25; −0.50)		−1.79 (−2.72; −0.66)	−1.48 (−2.79; −0.68)	−1.37 (−2.32; −0.52)
**HAZ**									
*n* (percent)	7 (2.69%)	1,071 (97.36%)	109 (41.44%)	69 (21.04%)	431 (90.17%)	0 (0%)	220 (72.61%)	136 (97.84%)	2,043 (69.63%)
Median (first; third quartile)	0.05 (−2.83; 3.88)	−2.67 (−3.59; −1.76)	−2.10 (−3.24; −1.10)	−2.23 (−2.94; −1.61)	−2.19 (−3.08; −1.19)		−2.73 (−3.64; −1.60)	−2.81 (−3.91; −1.92)	−2.56 (−3.48; −1.59)

**Figure 2:**
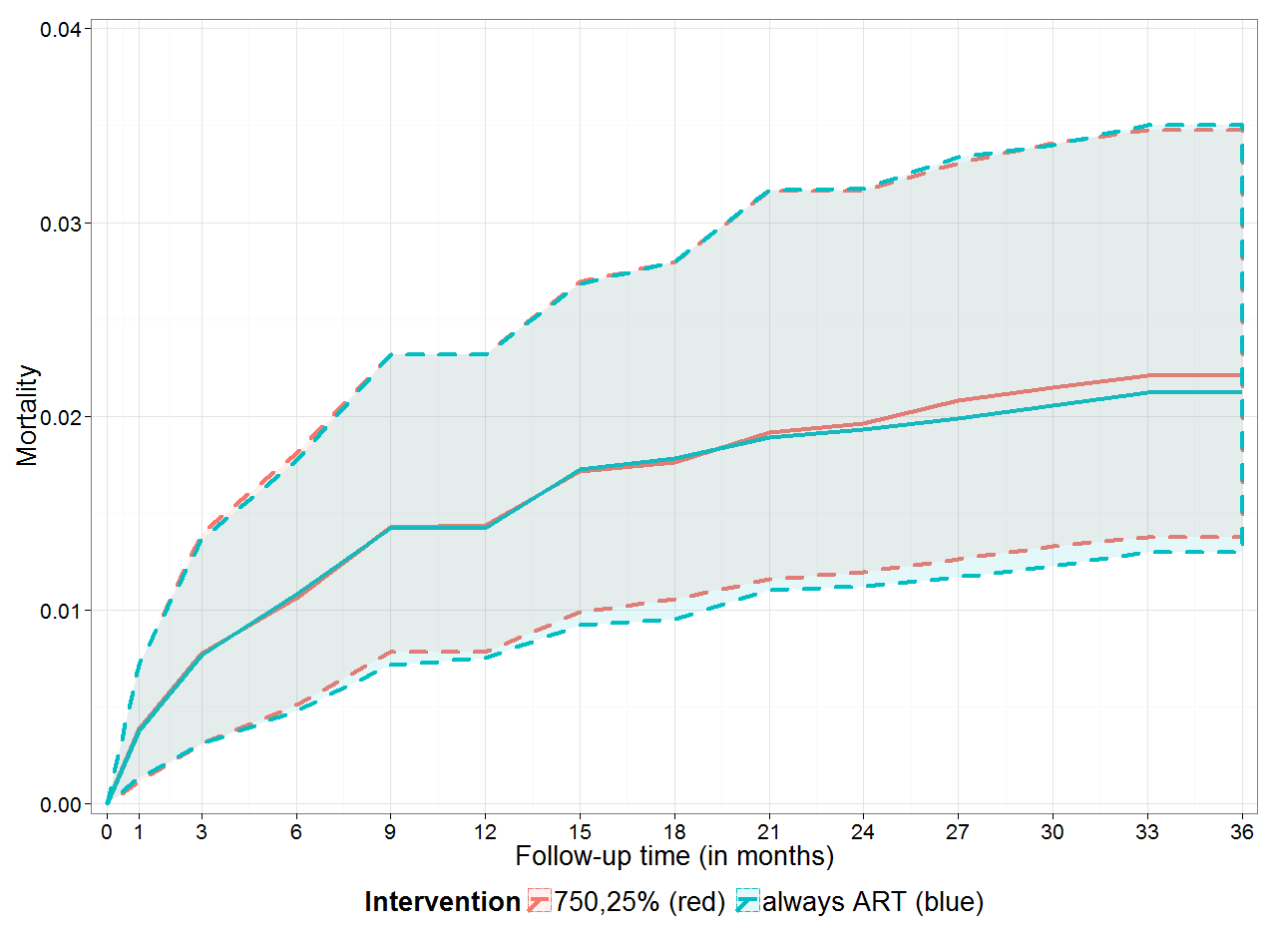
Estimated cumulative mortality for immediate versus deferred ART. Estimated cumulative mortality (including 95% bootstrap CI, dashed lines) over 3 y if ART was given irrespective of CD4 count and CD4% (blue line, “always ART”) and if ART was given if the CD4 count was below 750 cells/mm^3^ or the CD4% was below 25% (red line, “750,25%”).

The results of the g-computation analysis provided higher mortality point estimates for initiating ART at lower CD4 values or not initiating ART at all (Table 4). This pattern became evident from 2 y after the first visit onwards. There was no mortality difference between initiating ART immediately, irrespective of CD4 values, or starting ART according to the WHO 2010 threshold of CD4 count <750 cells/mm^3^ or CD4% <25%: estimates of cumulative mortality after 3 y of follow-up were 2.1% (95% CI: 1.3%–3.5%) and 2.2% (95% CI: 1.4%–3.5%), respectively. Figure 2 illustrates these estimates in detail, highlighting the almost identical estimated cumulative mortality (and 95% confidence intervals) for both ART initiation rules during the entire follow-up period.

**Table 4:** Estimated mortality (and 95% bootstrap confidence intervals) after 1, 2, and 3 y of follow-up for different initiation strategies.

Threshold	Years of Follow-Up		
	1 y	2 y	3 y
No ART	1.7% (0.9%–3.2%)	2.8% (1.7%–6.0%)	3.4% (2.1%–6.5%)
ART if CD4 count <250 cells/mm^3^ or CD4% <15	1.4% (0.8%–2.3%)	2.2% (1.4%–3.8%)	2.5% (1.6%–4.1%)
ART if CD4 count <500 cells/mm^3^ or CD4% <20	1.4% (0.8%–2.3%)	2.1% (1.3%–3.2%)	2.3% (1.4%–3.5%)
ART if CD4 count <750 cells/mm^3^ or CD4% <25	1.4% (0.8%–2.3%)	1.9% (1.2%–3.2%)	2.2% (1.4%–3.5%)
ART irrespective of CD4 count	1.4% (0.7%–2.3%)	1.9% (1.1%–3.2%)	2.1% (1.3%–3.5%)

The sensitivity analysis included 851 patients, 245 of whom were LTFU. We were able to link 32 (13.1%) patients LTFU to vital registration data for the Hlabisa HIV Treatment and Care Programme, Khayelitsha ART Programme, and McCord Hospital cohorts, and found three to be dead (one per cohort). After 3 y of follow-up the percentage of children who died was estimated as 6% (95% CI: 4.7%–7.4%) for children who received ART immediately irrespective of their CD4 count, 5.9% (95% CI: 4.6%–7.4%) for children starting ART at CD4 count <750 cells/mm^3^ or CD4% <25%, and 7.8% (95% CI: 5.1%–10.4%) for children receiving no ART at all.

**Figure 3:**
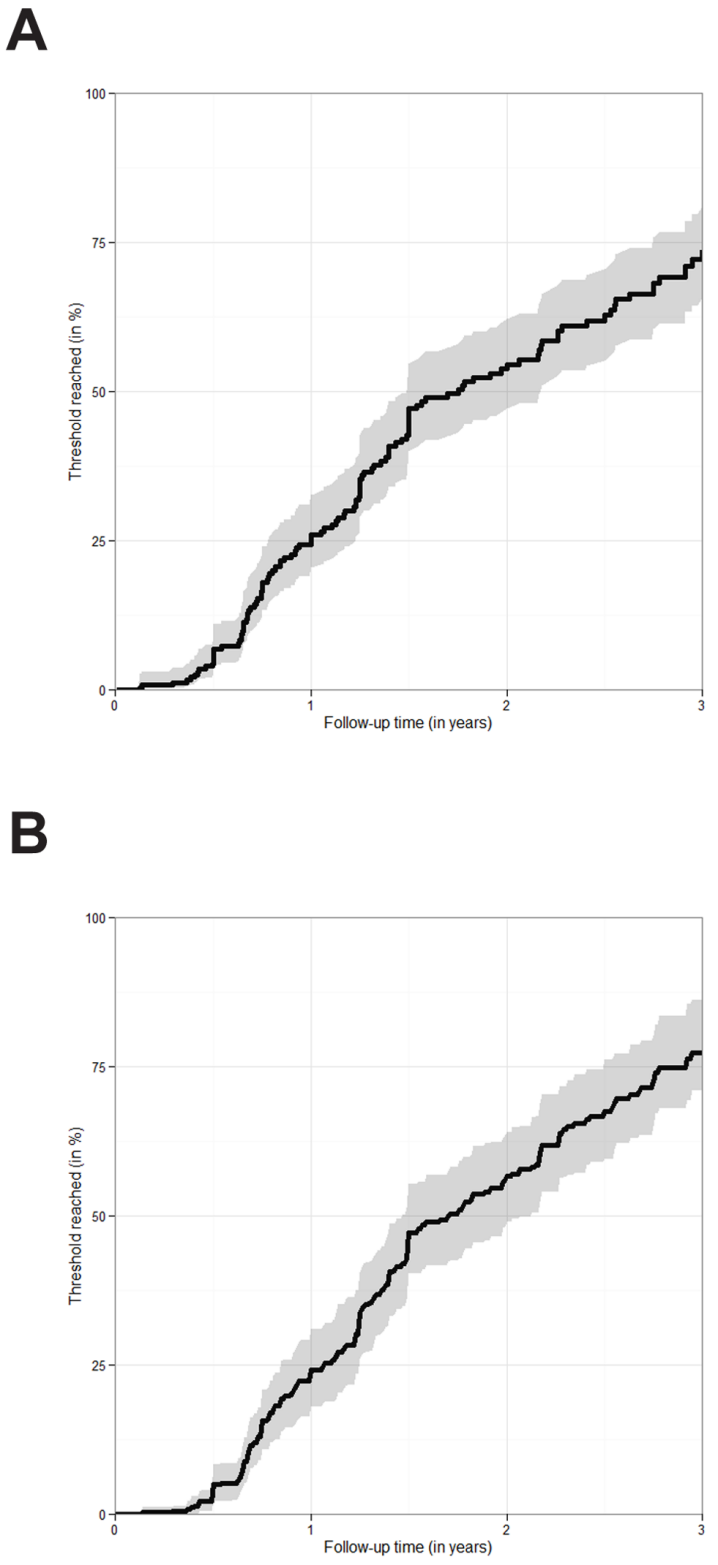
Estimated probability of falling below a CD4 count of 750 cells/mm^3^ or a CD4 percentage of 25%. (A) Time to fall below the CD4 threshold of a CD4 count of 750 cells/mm^3^ or a CD4% of 25% estimated via the Kaplan-Meier estimator. The figure is based on 322 children presenting with a CD4 count of 750 cells/mm^3^ or above and a CD4% of 25% or above. Only pre-ART follow-up is considered. 95% confidence intervals are visualised via the grey shaded area. (B) Time to fall below the CD4 threshold of a CD4 count of 750 cells/mm^3^ or a CD4% of 25%. The figure is based on 322 children presenting with a CD4 count of 750 cells/mm^3^ or above and a CD4% of 25% or above. ART initiation, death, and LTFU were treated as competing risks. The probability of falling below the threshold was estimated via the cumulative incidence of falling below the threshold before any other event occurred divided by one minus the probability that any other event occurred before the threshold was reached. 95% confidence intervals were obtained via bootstrapping and are visualised via the grey shaded area.

When considering the pre-ART data of the 322 children (11% of all children) who presented with a CD4 count above 750 cells/mm^3^ and CD4% above 25%, an estimated 26.0% (95% CI: 20.4%–32.7%) fell below this threshold after 1 y, 54.5% (47.2%–62.2%) after 2 y, and 72.2% (64.3%–79.6%) after 3 y (Figure 3A). The estimated probabilities of falling below the threshold were slightly higher when ART initiation, death, and LTFU were treated as competing events (Figure 3B).

## Discussion

### Statement of Principal Findings

Our results indicate no mortality difference for up to 3 y between immediate ART initiation irrespective of CD4 values and deferred ART initiation using a CD4 threshold of CD4 count <750 cells/mm^3^ or CD4% <25%, though point estimates for mortality were higher when ART was initiated at lower CD4 values (or not at all).

### Strengths of the Study

To our knowledge, this is the first attempt to estimate the effect on mortality of starting ART at different CD4 values in children aged 2–5 y. Both RCTs and previous causal modelling studies of observational data included a wider age range of children, with relatively small numbers of children in the 2–5-y age group [6],[9],[28],[29]. Although post hoc subgroup analyses of the RCT data restricted to this age range have been conducted, results were inconclusive [30]. Moreover, the observational studies had few children without either clinically or immunologically severe disease and did not compare different CD4 thresholds but rather concentrated on the effect of ART itself [28],[29].

Our analysis included a large dataset of nearly 3,000 children with relatively long follow-up from three resource-limited countries with high HIV burden. Because of the good availability of CD4 and WAZ data, which is unusual in this setting, as well as the use of g-computation, we were able to adjust for measured time-dependent confounding of CD4 count, CD4%, and WAZ. In addition, we accounted for missing data using multiple imputation.

The use of a causal modelling technique was essential to accurately estimate mortality associated with starting ART at different CD4 values in the presence of time-dependent confounding by indication, whereby the illest children have the greatest risk of mortality and are also most likely to be started on ART. There was clear evidence for time-dependent confounding: a naïve analysis of the data simply adjusting for demographic and disease severity characteristics at enrolment showed that ART initiation was associated with higher mortality than no ART initiation (hazard ratio = 1.80; 95% CI: 1.03–3.15). Other causal modelling techniques such as inverse probability of treatment weighting, g-estimation, or targeted maximum likelihood estimation could also have been used to derive results that adjust for time-dependent confounding, though g-computation is probably the most intuitive when dealing with dynamic treatment comparisons [13].

### Limitations

Although we used causal modelling, this analysis is based on observational data, and the results may be affected by residual confounding. For example, WHO clinical stage data were not routinely available throughout follow-up, and so we may not have adequately adjusted for the effect of clinical disease severity. We addressed this possibility by adjusting for WAZ, which is a surrogate of disease severity and should at least partly capture staging information [23]. Some stage-defining events such as HIV encephalopathy or HIV-associated nephropathy may, however, not be reflected by WAZ.

When interpreting estimates of mortality associated with starting ART at high CD4 values, it should be noted that relatively few children initiated ART before their CD4 count or CD4% declined. Since country and WHO guidelines at the time did not recommend ART initiation at high CD4 values unless children had WHO clinical stage 3 or 4 disease, it is plausible that these children may have initiated therapy on the basis of advanced clinical disease. The median WAZ in children starting ART with CD4 >750 cells/mm^3^ or CD4% >25% was −1.42 (Table 1), indicating that a proportion of these children had clinically severe disease. Our analysis may therefore underestimate mortality reductions associated with starting ART at high CD4 values in a child without symptomatic disease. Nevertheless, our mortality estimates associated with immediate ART initiation or starting ART at a high CD4 value are consistent with the probability of experiencing AIDS or death found in both the immediate and deferred ART arms of the PREDICT trial [9]. It would be useful to repeat this analysis using data from other settings, for example, in high-income settings where more children start ART earlier in the course of their disease.

Further limitations of our study include not examining outcomes beyond 3 y of follow-up. This limitation is especially relevant with respect to evaluating toxicity risk and the effect of development of resistance in a context of limited second-line options. In addition, children included in our study were mostly from urban settings with facilities for electronic data collection in southern Africa, especially from South Africa. Generalisability to rural areas and sub-Saharan African countries with less well-resourced health care systems may be limited. In addition, country-specific differences regarding drug regimens, public health programmes, and breastfeeding [31] and its implications for the timing of HIV infection may contribute to both mortality figures and the success of different treatment strategies.

Our analysis was specifically targeted at examining the WHO 2010 guidelines for ART initiation for children aged 2–5 y and how mortality would be affected by the WHO 2013 guideline recommendations. There are, however, other interesting comparisons that remain unexplored: for example, we did not include children between 1 and 2 y of age. There are currently no data that show mortality benefits of early ART in this age group [3]; a comparison with the age groups in this analysis would therefore be useful.

### Mortality Estimates in Context

The absolute mortality estimates for all ART initiation strategies in our study are relatively low [10],[28]. This finding may partly be explained by the analysis being restricted to children aged at least 2 y at their first visit to an HIV care and treatment facility. Our data therefore comprise a cohort of survivors who have not died and have not been referred for HIV care or ART prior to reaching 2 y of age, and so may have less rapidly progressing disease [10],[11],[32]. However, it is precisely in this group of children that the optimal timing of ART initiation needs to be determined, as WHO 2010 guidelines already recommend immediate ART in children presenting at less than 2 y of age [16]. Moreover, our mortality estimates are comparable to the results of the PREDICT trial, which reported a 2.1% and 1.3% risk of death/AIDS events after 144 wk in the early and deferred ART arms, respectively [9].

We may still have underestimated mortality. Children with no visits after enrolment were excluded from the analysis, as at least one follow-up visit is needed in order for children to have the possibility to initiate ART and thus allow the implementation of our methodology. The excluded children had higher CD4 counts (median [first; third quartile]: 694 cells/mm^3^ [382; 1,073]) and CD4% values (median [first; third quartile]: 21% [13%; 28%]) but lower WAZ values (median [first; third quartile]: −1.9 [−0.7; −3.3]), and their mortality may be different from those included in the analysis.

In addition, among the 2,934 children included in the analysis, those LTFU were censored at their last visit, and mortality may also be higher among those LTFU compared to those remaining in care [18],[33],[34]. To address this potential problem we conducted a sensitivity analysis, linking data from three of the South African cohorts with the national vital registry and imputing survival times and events of children LTFU and unascertained with multiple imputation as described previously [26]. This analysis confirmed that mortality might be higher than reported, though the relative differences between the strategies in the sensitivity analysis after 3 y of follow-up remained similar to those in our initial results ignoring LTFU that yielded much lower mortality estimates. It is worth mentioning that our estimate of mortality for children receiving no ART (7.8% [95% CI: 5.1%–10.4%]) is consistent with estimates of mortality from previous studies among children in this age group in the absence of ART [10]. However, the linkage information we had was sparse, the sample size was small, and data were available only from South Africa, so mortality estimates derived after linking data with the death registry should be interpreted with caution.

### Implications and Considerations for Clinicians and Policymakers

The WHO guidelines changed in 2013 and now recommend ART initiation in all children less than 5 y of age irrespective of clinical or immunological disease severity. Our results suggest that this change in ART initiation criteria would neither reduce nor increase mortality. There may, however, be other considerations regarding the optimal timing of ART. For example, while the PREDICT trial showed that the immune response to ART was equivalent in both groups, children in the deferred group spent more time at a lower CD4 count, which may result in more chronic inflammation and a higher risk of clinical events. This consideration is especially important for the older children in our age group, as their thymic responses are not as good as in children under 2 y [35]. However, there was no difference in the number of CDC category B or C events between the two arms in the PREDICT trial [8],[9]. The long-term effects of earlier ART initiation on immune function, growth, neurological development, toxicity, and development of resistance remain to be explored. In addition, maintaining long-term adherence can be particularly challenging in young children because of the need for a caregiver to administer medication and possible caregiver changes, lack of child-friendly formulations, and the need for frequent dose changes in growing children.

Programmatically it may be simpler to have a single treatment initiation approach for all children <5 y of age that does not require regular pre-ART follow-up and CD4 measurement, especially in settings where access to CD4 measurement is limited [36]. Coordination of recommended regimen choices with age thresholds for starting ART would also ease implementation. In our study only about 11% of children aged 2–5 y had CD4 count >750 cells/mm^3^ and CD4% >25% at presentation, and we estimated 72.2% (see Figure 3A) to have progressed below these values within 3 y, though the proportion might be even larger during early follow-up, given that children who were censored because they were LTFU or started ART may have had faster disease progression than others. Deferring treatment until CD4 count drops below 750 cells/mm^3^ or CD4% drops below 25% may not delay the onset of therapy very much, and may risk a child becoming LTFU and/or not having his/her repeat CD4 measured, thus not starting on treatment promptly after dropping below the initiation threshold. However, it would be important to ensure that immediate ART initiation in children aged 2–5 y does not happen at the expense of prioritising early infant diagnosis and rapid initiation, which has been shown to reduce mortality but may be more challenging to implement. This consideration is especially important in resource-limited settings, where an increasing number of patients often overburdens health care facilities, and clinics are therefore not able to trace children who miss their infant diagnostic testing and results or CD4 testing appointments [37],[38].

It has also been suggested that early ART initiation may reduce the high loss to follow-up frequently observed in pre-ART care [39]. However, loss to follow-up has not consistently been shown to be lower in children commenced on ART compared to those not on ART [40]. In addition, there may be selection bias, as children more likely to remain in care are more likely to get started on therapy.

### Conclusion

Our results indicate that in children aged 2–5 y in southern Africa, there is no difference in mortality between starting ART immediately and deferring until CD4% drops below 25% or CD4 count drops below 750 cells/mm^3^. The overall clinical and programmatic implications of earlier ART initiation, such as effects on morbidity-related outcomes, retention in care, cost-effectiveness, adherence, and drug resistance, remain to be explored.

## Abbreviations

ART: antiretroviral therapy
CD4%: CD4 percentage
CDC: US Centers for Disease Control and Prevention
HAZ: height-for-age *z*-score
IeDEA-SA: International Epidemiologic Databases to Evaluate AIDS–Southern Africa
LTFU: lost to follow-up
PREDICT: Pediatric Randomized to Early versus Deferred Initiation in Cambodia and Thailand
RCT: randomized controlled trial
WAZ: weight-for-age *z*-score
WHO: World Health Organization
